# Pilot Exploratory Analysis of Serum Gonadal Hormones, Inflammatory Proteins, and Intracerebral Hemorrhage Outcomes

**DOI:** 10.3390/ijms26178334

**Published:** 2025-08-28

**Authors:** Yisi Ng, Wenjing Qi, Anna Covington, Bobby Boone, Cynthia Kuhn, Andrew B. Nixon, Nicolas Kon Kam King, Peter F. Kranz, Thomas Christianson, Roshni Thakkar, Daniel T. Laskowitz, Cina Sasannejad, Miran Bhima, Vijay Krishnamoorthy, Shreyansh Shah, Amy K. Wagner, Michael L. James

**Affiliations:** 1 Duke-NUS Medical School, Singapore 169857, Singapore; yisi.91@gmail.com (Y.N.); daniel.laskowitz@duke.edu (D.T.L.); 2 Brain Injury Translational Research Center, Duke University, Durham, NC 27710, USA; 3 Data Coordinating Center, University of Utah, Salt Lake City, UT 84112, USA; wenjing.qi@duke.edu (W.Q.);; 4 Department of Anesthesiology, Duke University, Durham, NC 27710, USA; annacovington21@gmail.com (A.C.); miran.bhima@duke.edu (M.B.); vijay.krishnamoorthy@duke.edu (V.K.); 5 Duke Institute for Brain Sciences, Duke University, Durham, NC 27710, USA; cynthia.kuhn@duke.edu; 6 Department of Pharmacology and Cancer Biology, Duke University, Durham, NC 27710, USA; 7 Duke Cancer Center, Duke University, Durham, NC 27710, USA; andrew.nixon@duke.edu; 8 Neuro Asia Care, Mount Elizabeth Hospital, Singapore 228510, Singapore; nicolas.k@neuroasiacare.com; 9 Department of Radiology, Duke University, Durham, NC 27710, USA; peter.kranz@duke.edu; 10 Department of Anesthesiology, University of Tennessee, Knoxville, TN 37920, USA; tchristianson@utmck.edu (T.C.); rthakkar@utk.edu (R.T.); 11 Department of Neurology, Duke University, Durham, NC 27710, USA; cina.sasannejad@duke.edu (C.S.); shreyansh.shah@duke.edu (S.S.); 12 Duke Clinical Research Institute, Duke University, Durham, NC 27715, USA; 13 Department of Physical Medicine & Rehabilitation, Neuroscience, and Clinical & Translational Science Institute, University of Pittsburgh, Pittsburgh, PA 15213, USA; wagnerak@upmc.edu

**Keywords:** intracerebral hemorrhage, antihypertensive, multiple regression, nicardipine

## Abstract

Intracerebral hemorrhage (ICH) remains the least treatable form of stroke, with inflammation implicated as a major pathophysiological feature. Hence, this study sought to associate serum proteins and hormones associated with inflammation and ICH outcomes. Patients presenting to Duke University Hospital with computed tomography-verified spontaneous, supratentorial, non-traumatic ICH within 24 h of symptom onset were prospectively recruited. In this pilot study, equal numbers of men and women and Black and White individuals were included and matched by a 6-month modified Rankin Score (mRS). The primary analyses were the correlation of L-ratios (LR; Log2(Day 2/Day 1 concentrations)) of serum gonadal hormones and neuroinflammatory proteins with mRS > 3 at 6 months. A total of 40 participants were included in this pilot study. LRs were significantly higher for C-reactive protein (CRP; *p* = 0.013) and lower for interleukin-6 (IL-6; *p* = 0.026) and surfactant protein-D (*p* = 0.036) in participants with unfavorable outcomes at 6 months after ICH. Further, higher CRP (*p* = 0.02) and lower IL-6 (*p* = 0.035) and surfactant protein-D (*p* = 0.041) LRs were associated with mRS > 3 at 6 months after ICH in multiple logistic regression analyses, adjusted for race and sex. The relationship amongst gonadal hormones, neuroinflammatory proteins, and ICH outcome is complex. In this pilot study, unfavorable outcomes after ICH may have been associated with selected inflammatory biomarkers. A larger scale study is warranted to define interactions between hormones, proteins, and their effects on ICH outcomes.

## 1. Introduction

Intracerebral hemorrhage (ICH), the second most common stroke subtype accounts for 10 to 30% of all stroke hospital admissions in the United States and is associated with a mortality of over 40% [1,2]. Among people who survive, only half regain functional independence within three months, and ICH remains the least treatable form of stroke, lacking any effective therapy [3]. Further, no blood biomarker has been studied sufficiently for guiding ICH diagnosis and management [4]. In preclinical and translational models of ICH, evidence exists for serological inflammatory proteins and gonadal hormones as potential prognostic indicators and therapeutic targets [5,6,7].

Although ICH clinical trials have historically focused on the reduction of hematoma volume, recent studies have identified adverse relationships between both neuroinflammation and systemic inflammation on neurological outcomes in ICH patients [2,3,8,9]. Additionally, elevated inflammatory proteins on admission have been associated with unfavorable outcomes for ICH patients [10,11,12,13,14]. Similarly, gonadal hormones have well-known associations with both inflammatory markers and neurological outcomes after acute injury [15,16], and several clinical trials have assessed gonadal hormones as potential therapeutics after acute brain insults [17,18]. Thus, better understanding of interactions between gonadal hormones, systemic inflammation, and neurological recovery may make neuroinflammatory mediators attractive prognostic and therapeutic targets.

This project seeks to employ exploratory analyses to identify key relationships between acute serum inflammatory proteins, gonadal hormones, and neurobehavioral outcomes in patients with ICH.

## 2. Results

Baseline characteristics were compared between favorable (mRS = 0–3) and unfavorable (mRS = 4–6) outcome groups in Table 1, with hematoma volume being the only variable of significant difference. Table 2 shows the concentration values of serum gonadal hormones and neuroinflammatory proteins on Day 1 and Day 2 after ICH, stratified by favorable and unfavorable 6-month mRS. Notable differences were seen in progesterone, CRP, and IL-6.

To test correlations between gonadal hormones and neuroinflammatory proteins, the Spearman correlation coefficient was assessed on Day 1 after ICH (Table S2). Significant correlations were found between estrogen-progesterone & -CRP, testosterone-MMP-3, Pai-1 active-Ang-2, & -TNF-α, and IL-6-progesterone & -CRP, setting a *p*-value of <0.01 as significant given multiple comparisons.

The distributions of serum gonadal hormone and neuroinflammatory protein concentrations on Day 1 (Figure S1), Day 2 (Figure S2), and FC (Figure S3) were found to be non-normative. Thus, LR (Figure S4) was used to address differences in change in the first 48 h after ICH. A two-sample comparison of hormone and protein LR and FC for favorable versus unfavorable 6-month mRS is presented in Table 3. Significant differences between participants with favorable and unfavorable outcomes were found for CRP, IL-6, and Surfprotd.

Multiple logistic regression analysis, adjusted for race and sex, found that CRP, IL-6, and Surfprotd LRs were associated with mRS > 3 at 6 months after ICH (Table 4, with FC for comparison). Using the same model, associations between gonadal hormone and neuroinflammatory protein concentrations on Day 1 or 2 with unfavorable 6-month outcomes were assessed, but no significant associations were found (Table S3). Finally, an enhanced multivariate regression model adjusted for race, sex, age, body mass index, history of hypertension, hematoma location and volume, and the presence of IVH was performed. In this model, no associations between blood gonadal hormones or neuroinflammatory protein concentrations were found with unfavorable mRS at 6 months after ICH.

Finally, trajectory analyses were performed to test associations between serum gonadal hormones and inflammatory proteins on the positive recovery trajectory over 6 months after ICH. Day 1 IL-6 and Day 2 CRP were initially significant, with higher concentrations associated with poorer outcomes before adjustment for multiple testing. However, no biomarker remained statistically significant after correction for multiple comparisons (Table 5).

## 3. Discussion

This exploratory study represents the first attempt to investigate correlations between acute changes in gonadal hormones, neuroinflammatory proteins, and clinical outcomes up to 6 months after ICH. In this cohort, we observed potentially interesting correlations between various hormones and proteins and possible associations between neuroinflammatory proteins and clinical outcomes in adjusted multivariate models. However, these associations failed to reach significance when adjusted for multiple comparisons. Interestingly, despite previous observations that sex is associated with outcomes after ICH [19,20], gonadal hormones failed to associate with clinical outcomes after ICH in our analyses.

Although various peripheral blood proteins have potential for diagnosis, therapeutic monitoring, risk stratification, or targets for intervention in the setting of acute ICH, none are currently used for clinical management. For example, both IL-6 and CRP are associated with neuroinflammation [21,22]; prior observations have suggested that IL-6 serum concentrations are an independent predictor of early hematoma growth and associated with poor 3-month outcomes, whereas CRP is independently associated with increased 30-day mortality post-ICH [23,24]. Similarly, in the present exploratory analyses, CRP and IL-6 concentrations correlated with each other and with dichotomized mRS at 6 months after ICH even when adjusting for covariates. Thus, the present study adds further weight to the importance of these neuroinflammatory proteins in acute mediating outcome after ICH. However, current human data regarding the modifiability of IL-6 and CRP expression as an intervention affecting ICH outcome are limited [25].

One novel finding from this study is the potential role of Surfprotd in ICH. A multimeric collection discovered in pulmonary epithelia and expressed in the brain [26], Surfprotd is involved in innate immunity by exerting antimicrobial effects and inhibiting inflammation [27,28]. While differential expression patterns in the brain have been investigated, the true role of CNS Surfprotd remains unclear [29], though modulating CNS inflammation has been implicated [30]. Two potential mechanisms have been proffered: interaction with toll-like receptors and CD14 to modulate microglia [31,32] and S-nitrosylation to induce chemotaxis and MAPK phosphorylation in the CNS [26,33]. In the present analyses, diminished Surfprotd expression in patients with worse outcomes may have been consistent with loss of anti-inflammatory protection.

While gonadal hormone effects have been studied extensively in preclinical models of CNS trauma, their effects on post-ICH outcomes are only beginning to be understood. However, we found no correlation between serum gonadal hormone measurements and outcome after ICH in our sample. This may be attributed to a lack of power or incomplete understanding of complex female gonadal hormone interactions with inappropriate measurement comparisons. Interestingly, progesterone is known to have pleiotropic CNS effects involving coagulation and inflammation that result in both injurious and beneficial effects [34,35]. Prior studies have begun to link IL-6, progesterone, and neurological recovery [36], and the present exploratory study seems to support these relationships. Notably, this study’s analyses did not identify gender-based covariation in these hormones; estradiol and progesterone were not uniquely protective or deleterious in female patients, nor was testosterone in male patients.

When analyzing multiple time-point measurements of a biological system, we chose to use FC, as the ratio provided a relative measure that could be used to compare the change between the two longitudinal conditions. Similarly, LR was calculated by taking the logarithm of the FC ratio, thus representing multiplicative change between two values rather than the absolute difference. Because FC was non-normative, we chose LR as our primary model. Ultimately, the use of LR over FC, Day 1, or Day 2 concentrations made little difference in terms of the observed correlations between any biomarker and 6-month mRS, and both measures are not well-suited for immediate clinical interpretation and applicability.

Finally, trajectory analyses were constructed to leverage the power of serial sampling in this cohort. While not reaching statistical significance for a positive outcome trajectory before the multiple comparison correction, both Day 1 IL-6 and Day 2 CRP were associated with negative trajectories, indicating that a higher concentration contributes to poor trajectory outcomes. These findings are consistent with our prior regression modeling. Additionally, given the exploratory nature of the study, all biomarkers with a *p*-value lower than 0.2 might be worth investigating for clinical significance in a larger cohort.

This study’s limitations include a relatively small sample, a short duration of serological monitoring, a lack of controls without ICH, and multiple statistical comparisons. Given the small stratified pilot sample in the current study, statistical models have a high probability of selection bias, over-fitting, and limited generalizability; thus, a larger sample would allow for adjustment for more recovery-related covariates, including hematoma location, and permit analyses across diverse subpopulations—for example, young Black females compared to older Hispanic males. Importantly, limiting the timeframe of the study to 48 h after ICH allowed for the investigation of the acute phase of injury; however, since the true time course of these hormones and proteins is unclear after ICH, even the relatively minor variability in the sampling of the present study (±4 h) has unclear effects. Observing frequent, standardized temporal changes in serum concentrations over several days or weeks would shed light on the body’s response to ICH. Similarly, serial standardized imaging timing coupled with serum biomarker concentrations would provide powerful correlations between serologic and radiologic biomarkers and physiology after ICH. Without reference to controls or a clinical control range, it is difficult to make inferences beyond statistical correlations and associations. A diverse array of other relevant clinical outcomes should be included in future studies. Although there appears to be no directional relationship between the human gonadal hormone and neuroinflammatory biomarker levels, complex relationships and interactions remain to be defined. Additionally, treating mRS as an ordinal variable rather than a dichotomized outcome may provide more descriptive results of the relationships between individual trajectories and biomarkers of interest. Finally, because our aim was to generate hypotheses from a pilot exploratory study, we did not correct for multiple comparisons across all of the statistical analyses. Future studies will need to correct for this before definite conclusions can be drawn.

Taken together, the present findings suggest the potential relevance of IL-6 and CRP in post-ICH recovery, warranting further investigation. Additionally, several biomarkers (e.g., Surfprotd, VEGF, Ang-2) were identified with demonstrated adjusted *p*-values < 0.2 and may be worth exploring in future studies. To pursue biomarker development, a sufficiently large sample size with serial sampling and imaging beyond the first few days after ICH would provide more powerful and informative analyses.

## 4. Materials and Methods

### 4.1. Study Population

All study procedures were approved by the Duke University Institutional Review Board. Patients from the emergency room, neurological ICU, and stroke ward were screened daily for potential ICH cases, including those with uncertain diagnoses. Informed consent was obtained from each enrolled subject or their legally authorized representative if the patient was unable to consent due to impaired capacity, assessed by an informed consent comprehension questionnaire.

### 4.2. Participants

From January 2009 to December 2015, all patients aged 18 years and above who presented to Duke University Hospital with an acute focal neurological deficit and computed tomography (CT)-verified spontaneous, non-traumatic ICH within 24 h of symptom onset (or last known well-time) were approached for consent. Of those approached, 182 patients were enrolled in the single-center parent observational study of ICH, from which a pilot cohort of 10 white female subjects, 10 black female subjects, 10 white male subjects, and 10 black male subjects were analyzed. Of these, an equal distribution of patients by race and gender had an mRS at 6 months post-ICH of 0 to 3 (defined as a favorable outcome) and 4 to 6 (defined as an unfavorable outcome). Patients with secondary causes of ICH, pregnant females (as verified by review of the standard of care urine pregnancy test), and patients with infratentorial hematoma locations were excluded from the study. Demographic and baseline data for each subject were obtained via chart abstraction (Table 1).

### 4.3. Imaging

Neuroimaging review by the study neurologist (MLJ) of the initial CT scan for each subject confirmed the diagnosis of spontaneous ICH. Hematoma volumes on the diagnostic CT were measured using AnalyzePro version 1.0 (AnalyzeDirect, Inc., Stilwell, KS, USA) and confirmed by a blinded study neuroradiologist (PFK). The hematoma location was dichotomized into lobar and non-lobar.

### 4.4. Neurological Outcome

Outcome was defined as the modified Rankin Scale (mRS) 6 months post-ICH, obtained from follow-up phone interviews with patients and LARs [37]. Phone interviews were conducted by study staff in a standardized fashion after sufficient training by certified mRS assessors [38].

### 4.5. Blood Sampling

Each enrolled subject had 10 mL of whole blood collected at enrollment or 24 ± 4 h after their last known normal (i.e., Day 1) and 48 ± 4 h after their last known normal (i.e., Day 2) via venous puncture or by accessing existing central venous, arterial, or peripheral intravenous catheters. The samples were centrifuged at 2000× *g* for ten minutes at 4 °C, and the supernatant (i.e., plasma) was aliquoted into five 1 mL aliquots and stored in a −80 °C freezer for subsequent measurement of serum gonadal hormone and neuroinflammatory-associated protein concentrations. The latency from sample collection to hormone/protein analyses ranged from 1–5 years.

### 4.6. Measurement of Serum Gonadal Hormone Concentrations

For serum gonadal hormone measurement, Estradiol DA (MP Biomedicals, Solon, OH, USA, catalog number 07-138102), Progesterone DA (MP Biomedicals, Solon, OH, USA, catalog number 07-170102), and Testosterone DA (MP Biomedicals, Solon, OH, USA, catalog number 07-189102) kits were used. Reagents, samples, standards, and controls were brought to room temperature, and the required volumes were added to the corresponding numbered tubes. Radioactive hormones and specific antibodies were added, and tubes were vortexed, mixed, and then incubated in a water bath. After the incubation, precipitants were added, and tubes were vortexed, mixed, and incubated again. Subsequently, tubes were centrifuged and aspirated, and the precipitate was counted in a gamma counter.

### 4.7. Measurement of Serum Inflammatory Protein Concentrations

Inflammatory proteins were chosen based on existing literature [21,39,40,41,42,43,44,45,46]. Angiopoietin 2 (Ang-2), active Plasminogen Activator Inhibitor-1 (Pai-1 active), total Plasminogen Activator Inhibitor-1 (Pai-1 total), Receptor for Advanced Glycation Endproducts (RAGE), C-reactive protein (CRP), matrix metalloproteinase 1, 3, and 9 (MMP-1, MMP-3, MMP-9), interleukin 6 and 8 (IL-6, IL-8, tumor necrosis factor-alpha (TNF-α), vascular endothelial growth factor (VEGF), and Surfactant protein-D (Surfprotd) were measured from the same serum samples used for gonadal hormone measurements. All samples were analyzed in duplicate to quantify protein expression using immunoassays. Assays used to measure the biomarkers are tabulated in Table S1. All assays were performed according to the individual manufacturer’s instructions.

### 4.8. Statistical Analyses

Analyses for this study were performed using the Statistical Analysis System (SAS^®^) software version 9.4 (Cary, NC, USA), University Edition, and R version 3.5.2. All subject/sample datasets were complete with no cases of missing data.

Two sample tests were performed to compare baseline characteristics between favorable (i.e., mRS = 0–3) and unfavorable (i.e., mRS = 4–6) 6-month neurological outcome groups. Continuous variables including age, body mass index (BMI), and hematoma volume were compared by two-sample *t*-tests (if passed normality tests) or Wilcoxon rank-sum tests (if failed normality tests). Binary variables such as past medical history of hypertension, intraventricular hemorrhage (IVH), and lobar hematoma location were compared by chi-squared tests (if all expected cell counts were greater than 5) or Fisher’s exact tests (if any expected cell count was less than 5).

In addition, fold change (FC; [Day 2]/[Day 1]) and logarithmic ratios (LR; Log2([Day 2]/[Day 1])) were calculated for serum gonadal hormone and inflammatory protein concentrations to compare changes in concentration over the first two days after ICH. FC and LR are often used when analyzing multiple measurements of a biological system taken at different times, as the change described by the ratio between time points may be easier to interpret than the difference. Day 1 and Day 2 concentrations, FC, and LR were compared between outcome groups (favorable and unfavorable 6-month mRS) by two-sample *t*-tests (if passed normality tests) or Wilcoxon rank-sum tests (if failed normality tests).

To evaluate the relationships between serum gonadal hormones and inflammatory proteins, Spearman correlation coefficients were computed for Day 1 concentrations. To assess the relationships between biomarkers and clinical outcomes, multiple logistic regression analyses were conducted across outcome groups (favorable and unfavorable 6-month mRS) for each serum gonadal hormone and inflammatory protein LR. Since FC was not normally distributed, LR was used as the primary analysis. One set of multiple logistic regression analyses was adjusted for race and sex; a second set of analyses was adjusted for race, sex, age, BMI, hypertension, location of ICH, ICH volume, and IVH as covariates.

Finally, trajectory analyses were performed to identify biomarkers associated with 6-month recovery trajectories in patients with ICH. The primary outcome variable, trajectory, was a binary variable. A favorable 6-month mRS, or positive trajectory, was coded as 1 in the data set, while an unfavorable 6-month mRS, or negative trajectory, was coded as 0. Although trajectories may be analyzed using PROC TRAJ or PROC GLIMMIX, PROC LOGISTIC was the most efficient way to analyze our data, given the binary nature of the primary outcome. PROC LOGISTIC was run using stepwise selection, with the descending option used because the positive trajectory was 1. The entry level was 0.05, and the exit level was 0.10. Due to a low sample size of 40 patients and with 32 independent variables, 16 logistic models were used to test each biomarker on both days per model. Once each model was tested and estimates with *p*-values were produced, the Benjamini–Hochberg (BH) procedure at 0.2 was used to account for the numerous models.

## 5. Conclusions

Interactions amongst gonadal hormones, neuroinflammatory proteins, and ICH pathophysiology are complex. Unfavorable outcomes after ICH may be associated with increases in CRP, IL-6, and Surfprotd in the first day after ICH. Surfprotd requires further study to understand biological implications in ICH. Gonadal hormone relationships with ICH recovery likely require subpopulation study and analyses of inter-hormone interactions.

## Figures and Tables

**Table 1:** Demographics and baseline characteristics.

	mRS 0~3	mRS 4~6
Age, years, mean (SD)	61.40 (12.42)	63.15 (12.79)
BMI, mean (SD)	30.04 (8.07)	29.17 (7.97)
Hematoma volume, cm^3^, mean (SD)	14.72 (16.28)	43.70 (33.19)
Past medical history of hypertension, N (%)	14	17
Intraventricular bleeding, N (%)	6	7
Lobar ICH, N (%)	10	10
Non-lobar ICH, N (%)	10	10

BMI: Body Mass Index; %: percentage; cm^3^: centimeters cubed; ICH, intracerebral hemorrhage, mRS: modified Rankin Scale at 6 months after intracerebral hemorrhage; N: number; SD: standard deviation; Hematoma volume *p*-value = 0.003.

**Table 2:** Differences in Day 1 and Day 2 mean (standard deviation) serum gonadal hormone and inflammatory protein concentrations between patients with favorable vs. unfavorable outcomes at 6 months after ICH.

	Day 1			Day 2		
	mRS 0~3	mRS 4~6	*p*-Value	mRS 0~3	mRS 4~6	*p*-Value
Estrogen	39.67 (62.80)	37.90 (21.26)	0.112	30.00 (34.74)	34.97 (25.74)	0.160
Progesterone	0.37 (0.50)	0.72 (0.51)	**0.014**	0.26 (0.41)	0.47 (0.47)	**0.02**
Testosterone	1.71 (1.76)	1.97 (1.83)	0.402	1.32 (1.23)	1.57 (1.40)	0.695
Ang-2	418.17 (309.67)	619.21 (702.89)	0.409	425.76 (261.81)	784.73 (764.90)	0.091
Pai-1 active	12,436.83 (16,690.28)	17,272.00 (16,639.43)	0.12	8861.78 (6522.37)	11,010.15 (7451.09)	0.285
Pai-1 total	28,682.40 (13,281.28)	31,645.50 (23,096.07)	0.882	26,032.70 (14,221.70)	23,096.07 (14,121.35)	0.925
RAGE	382.65 (788.33)	496.87 (580.41)	0.06	662.02 (1610.11)	725.16 (724.25)	0.19
CRP Mean (SD)	2.44 (4.38)	2.96 (1.95)	**0.012**	3.51 (5.35)	9.7 (8.11)	**<0.001**
MMP-1	5.14 (4.02)	6.43 (5.81)	0.715	5.28 (4.81)	6.28 (4.36)	0.337
MMP-3	19.43 (16.77)	21.69 (15.32)	0.457	35.1 (46.53)	35.8 (29.14)	0.273
MMP-9	147.97 (121.8)	137.39 (81.53.)	0.839	121.25 (80.34)	132.08 (60.68)	0.508
IL-6	4.40 (5.00)	17.83 (24.66)	**<0.001**	4.93 (4.45)	10.55 (12.10)	**0.005**
IL-8	7.72 (6.41)	9.99 (5.31)	0.039	6.79 (6.41)	9.11 (5.28)	0.12
TNF-α	2.45 (1.86)	2.77 (1.97)	0.285	2.38 (1.62)	2.91 (2.02)	0.351
VEGF	50.99 (22.29)	63.95 (30.67)	0.135	53.63 (22.45)	57.83 (26.64)	0.593
Surfprotd	9.38 (6.84)	7.59 (4.60)	0.607	9.39 (6.61)	6.56 (3.76)	0.107

Ang-2: Angiopoietin 2 (pg/mL); CRP: C-reactive protein (mg/dL); Day 1: serum concentration one day after ICH; Day 2: serum concentration two days after ICH; Estrogen (pg/mL); ICH: intracerebral hemorrhage; IL-6: interleukin 6 (pg/mL); IL-8: interleukin 8 (pg/mL); MMP-1: matrix metalloproteinase 1 (ng/mL); MMP-3: matrix metalloproteinase 3 (ng/mL); MMP-9: matrix metalloproteinase 9 (ng/mL); mRS: modified Rankin Scale; Pai-1 active: active Plasminogen Activator Inhibitor-1 (IU/mL); Pai-1 total: total Plasminogen Activator Inhibitor-1 (IU/mL); Progesterone (ng/mL); RAGE: Receptor for Advanced Glycation Endproducts (pg/mL); SD: standard deviation; Surfprotd: Surfactant protein-D (ng/mL); Testosterone (ng/dL); TNF-α: tumor necrosis factor alpha (pg/mL); VEGF: vascular endothelial growth factor (pg/mL). Bolded entries represented statistically significant findings.

**Table 3:** Differences in fold change and log ratio mean (standard deviation) of serum gonadal hormone and inflammatory protein concentrations between patients with favorable vs. unfavorable outcomes at 6 months after ICH.

	Log Ratio			Fold Change		
	mRS 0~3	mRS 4~6	*p*-Value	mRS 0~3	mRS 4~6	*p*-Value
Estrogen	−0.30 (1.27)	−0.23 (0.65)	0.825	1.15 (1.02)	0.89 (0.48)	0.922
Progesterone	−0.96 (1.68)	−0.73 (1.54)	0.66	0.89 (0.87)	0.98 (1.03)	0.684
Testosterone	−0.10 (1.01)	−0.41 (0.82)	0.294	1.18 (0.86)	0.87 (0.49)	0.409
Ang-2	0.13 (0.44)	0.39 (0.62)	0.13	1.15 (0.4)	1.43 (0.6)	0.19
Pai-1 active	−0.07 (1.23)	−0.48 (1.42)	0.331	1.32 (1.17)	1.23 (1.98)	0.239
Pai-1 total	−0.23 (0.6)	−0.07 (0.78)	0.675	0.92 (0.36)	1.14 (0.99)	0.675
RAGE	0.56 (0.68)	0.54 (1.11)	0.947	1.67 (1.01)	1.91 (1.45)	0.882
CRP	0.68 (1.12)	1.72 (1.39)	**0.013**	2.16 (1.83)	5.24 (5.53)	**0.022**
MMP-1	−0.09 (0.72)	0.12 (0.69)	0.882	1.04 (0.45)	1.265 (0.83)	0.882
MMP-3	0.61 (1.65)	0.70 (0.75)	0.561	1.9 (1.65)	1.85 (0.98)	0.561
MMP-9	−0.15 (0.73)	0.01 (1.12)	0.6	1.01 (0.48)	1.35 (1.19)	0.756
IL-6	0.4 (1.2)	−0.49 (1.23)	**0.026**	1.81 (1.65)	0.93 (0.61)	**0.026**
IL-8	−0.02 (0.85)	−0.14 (0.8)	0.715	1.17 (0.84)	1.02 (0.41)	0.715
TNF-α	0.02 (0.18)	0.04 (0.5)	0.913	1.03 (0.18)	1.09 (0.46)	0.882
VEGF	0.08 (0.51)	−0.14 (0.46)	0.156	1.12 (0.35)	0.95 (0.29)	0.108
Surfprotd	0.04 (0.36)	−0.20 (0.31)	**0.036**	1.06 (0.28)	0.89 (0.19)	**0.027**

Ang-2: Angiopoietin 2; CRP: C-reactive protein; ICH: intracerebral hemorrhage; IL-6: interleukin 6; IL-8: interleukin 8; MMP-1: matrix metalloproteinase 1; MMP-3: matrix metalloproteinase 3; MMP-9: matrix metalloproteinase 9; mRS: modified Rankin Scale; Pai-1 active: active Plasminogen Activator Inhibitor-1; Pai-1 total: total Plasminogen Activator Inhibitor-1; RAGE: Receptor for Advanced Glycation Endproducts; Surfprotd: Surfactant protein-D; TNF-α: tumor necrosis factor alpha; VEGF: vascular endothelial growth factor. Fold Change = serum concentration two days after ICH/serum concentration one day after ICH. Log ratio = Log2(serum concentration two days after ICH/serum concentration one day after ICH). Bolded entries represented statistically significant findings.

**Table 4:** Multiple logistic regression of fold change and log ratio for serum gonadal hormones and inflammatory proteins on dichotomized modified Rankin Scale (0–3 versus 4–6) at 6 months after intracerebral hemorrhage, adjusted for race and sex as covariates.

	Log Ratio			Fold Change		
	Point Estimate	95% CI	*p*-Value	Point Estimate	95% CI	*p*-Value
Estrogen	1.051	(0.527, 2.095)	0.888	0.606	(0.236, 1.557)	0.298
Progesterone	1.095	(0.732, 1.639)	0.659	1.134	(0.569, 2.257)	0.721
Testosterone	0.642	(0.300, 1.376)	0.717	0.446	(0.152, 1.309)	0.141
Ang-2	2.651	(0.759, 9.264)	0.127	3.205	(0.819, 12.536)	0.094
Pai-1 active	0.775	(0.470, 1.278)	0.319	0.963	(0.640, 1.447)	0.854
Pai-1 total	1.501	(0.550, 4.095)	0.428	1.776	(0.501, 6.298)	0.374
RAGE	0.976	(0.487, 1.956)	0.945	1.189	(0.695, 2.036)	0.527
CRP	2.037	(1.118, 3.711)	**0.02**	1.297	(0.987, 1.706)	0.062
MMP-1	1.615	(0.624, 4.176)	0.323	1.739	(0.568, 5.323)	0.333
MMP-3	1.181	(0.528, 2.640)	0.686	0.974	(0.604, 1.573)	0.916
MMP-9	1.308	(0.576, 2.967)	0.521	1.857	(0.705, 4.889)	0.21
IL-6	0.490	(0.253, 0.950)	**0.035**	0.365	(0.136, 0.979)	**0.045**
IL-8	0.830	(0.372, 1.848)	0.648	0.657	(0.226, 1.911)	0.441
TNF-α	1.099	(0.215, 5.620)	0.909	1.737	(0.248, 12.182)	0.579
VEGF	0.372	(0.095, 1.465)	0.158	0.184	(0.023, 1.481)	0.112
Surfprotd	0.073	(0.006, 0.894)	**0.041**	0.016	(<0.001, 0.822)	**0.04**

95% CI; 95% confidence interval; Ang-2: Angiopoietin 2; CRP: C-reactive protein; IL-6: interleukin 6; IL-8: interleukin 8; MMP-1: matrix metalloproteinase 1; MMP-3: matrix metalloproteinase 3; MMP-9: matrix metalloproteinase 9; mRS: modified Rankin Scale; Pai-1 active: active Plasminogen Activator Inhibitor-1; Pai-1 total: total Plasminogen Activator Inhibitor-1; RAGE: Receptor for Advanced Glycation Endproducts; Surfprotd: Surfactant protein-D; TNF-α: tumor necrosis factor alpha; VEGF: vascular endothelial growth factor; bolded *p*-values < 0.05. Fold Change = serum concentration two days after ICH/serum concentration one day after ICH. Log ratio = Log2(serum concentration two days after ICH/serum concentration one day after ICH). Bolded entries represented statistically significant findings.

**Table 5:** Trajectory analysis for selected inflammatory proteins (with *p*-value < 0.02) on positive recovery defined by dichotomized modified Rankin Scale (0–3) at 6 months after intracerebral hemorrhage.

	Intercept (*p*-Value)	Estimate	Chi-Squared	*p*-Value	BH Adjusted *p*-Value
IL-6, Day 1	1.61 (0.011)	−0.388	5.237	0.022	0.013
CRP, Day 2	1.19 (0.028)	<−0.001	4.884	0.027	0.025
CRP, Day 1	1.102 (0.065)	−0.15	3.654	0.065	0.038
Surfprotd, Day 2	1.048 (0.082)	−0.12	3.01	0.083	0.05
VEGF, Day 1	0.992 (0.116)	−0.1	2.49	0.114	0.063
Ang-2, Day 2	0.956 (0.147)	−0.08	2.1	0.147	0.075
TNF-α, Day 1	0.931 (0.169)	−0.07	1.87	0.169	0.088
MMP-1, Day 2	0.902 (0.189)	−0.06	1.75	0.189	0.1

Ang-2: Angiopoietin 2; BH: Benjamini–Hochberg correction for multiple comparisons; CRP; C-reactive protein; Day 1: serum concentration one day after ICH; Day 2: serum concentration two days after ICH; IL-6: interleukin 6; MMP-1: matrix metalloproteinase 1; Surfprotd: Surfactant protein-D; TNF-α: tumor necrosis factor alpha; VEGF: vascular endothelial growth factor; If a *p*-value is smaller than its BH-adjusted threshold, it is considered significant after correction.

## Data Availability

The original contributions presented in this study are included in the article/Supplementary Material. Further inquiries can be directed to the corresponding author(s). The raw data supporting the conclusions of this article will be made available by the authors on request.

## Supplementary Materials

The following supporting information can be downloaded at: https://www.mdpi.com/article/10.3390/ijms26178334/s1.
