# Supplementary material for: Pilot Exploratory Analysis of Serum Gonadal Hormones, Inflammatory Proteins, and Intracerebral Hemorrhage Outcomes
Source: Int J Mol Sci. 2025 Aug 28;26(17):8334. doi: 10.3390/ijms26178334 (PMC12427954; doi:10.3390/ijms26178334)
Supplement: Supplementary file 1 [file ijms-26-08334-s001.zip › ijms-3714083-supplementary.pdf]

Appendix A

Table A1. Assays used in the measurement of biomarkers.

Biomarker	Assay	Standard Assay Range	Sensitivity
Ang-2	CiraPlex human Ang-2 assay (Meso Scale Discovery, Rockville, MD, USA, Lot #197-5032)	10–10,000 pg/mL	10 pg/mL
PAI-1 active	Human PAI-1 active assay (Molecular Innovations, MI, USA, Lot #100-0204	0.625–20 IU/mL	0.6 IU/mL
PAI-1 total	Human PAI-1 total assay Molecular Innovations, MI, USA, Lot #100-0039)	0.1–100 ng/mL	0.5 ng/mL
RAGE	Human RAGE assay (Meso Scale Discovery, Rockville, MD, USA, Lot #100-0171)	10–2000 pg/mL	4 pg/mL
VEGF-A	Human VEGF assay (Sigma Aldrich, St. Louis, MO, USA, Catalog #L451RHA-1)	3–1000 pg/mL	4 pg/mL
CRP	Human CRP assay (Sigma Aldrich, St. Louis, MO, USA, Catalog #N451STB-1)	0.01–1 mg/dL	0.01 mg/dL
IL-6/TNF/IL-8	Proinflammatory panel 1 (Meso Scale Discovery, Rockville, MD, USA, Catalog #N05049A-1)	1–10,000 pg/mL	0.2–10 pg/mL
MMP-1/3/9	MMP 3-plex (Meso Scale Discovery, Rockville, MD, USA, Catalog #N45034A-1)	0.001–10 ng/mL	0.0083–0.025 ng/mL
Surfactant protein-D	R&D human SP-D assay (R&D Systems, Inc., Minneapolis, MN, USA, Lot #P109121)	0.63–40 ng/mL	0.37 ng/mL

Ang-2: Angiopoietin 2; CRP: C-reactive protein; IL-6: interleukin 6; IL-8: interleukin 8; MMP-1: matrix metalloproteinase 1; MMP-3: matrix metalloproteinase 3; MMP-9: matrix metalloproteinase 9; Pai-1 active: active Plasminogen Activator Inhibitor-1; Pai-1 total: total Plasminogen Activator Inhibitor-1; RAGE: Receptor for Advanced Glycation Endproducts; TNF: tumor necrosis factor alpha; VEGF-A: vascular endothelial growth factor.

Table A2. Spearman correlation of Day 1 serum gonadal hormones and inflammatory proteins at Day 1 after ICH, ǀrǀ (*p*-value).

	Estrogen	Progesterone	Testosterone	Ang-2	Pai-1 active	Pai-1 total	RAGE	CRP	MMP-1	MMP-3	MMP-9	IL-6	IL-8	TNF-α€	VEGF	Surfprot
Estrogen	1.000															
Progesterone	0.433<br>**(0.006)**	1.000														
Testosterone	0.356<br>(0.026)	−0.009<br>(0.956)	1.000													
Ang-2	0.271<br>(0.096)	0.221<br>(0.170)	0.144<br>(0.375)	1.000												
Pai-1 active	0.397<br>(0.012)	0.261<br>(0.104)	0.295<br>(0.064)	0.408<br>**(0.009)**	1.000											
Pai-1 total	0.161<br>(0.329)	0.144<br>(0.375)	0.151<br>(0.353)	0.111<br>(0.494)	0.556<br>**(<0.001)**	1.000										
RAGE	−0.193<br>(0.240)	0.207<br>(0.201)	0.003<br>(0.987)	0.163<br>(0.315)	0.166<br>(0.305)	−0.142<br>(0.383)	1.000									
CRP	0.432<br>**(0.006)**	0.393<br>(0.012)	0.356<br>(0.024)	0.379<br>(0.016)	0.383<br>(0.015)	0.133<br>(0.414)	0.110<br>(0.498)	1.000								
MMP-1	−0.119<br>(0.471)	0.146<br>(0.370)	−0.129<br>(0.429)	0.102<br>(0.531)	0.161<br>(0.320)	0.399<br>(0.011)	0.058<br>(0.724)	−0.155<br>(0.340)	1.000							
MMP-3	0.170<br>(0.300)	−0.060<br>(0.711)	0.497<br>**(0.001)**	0.202<br>(0.212)	0.061<br>(0.707)	−0.010<br>(0.955)	0.171<br>(0.291)	0.265<br>(0.098)	−0.191<br>(0.239)	1.000						
MMP-9	0.351	0.318	0.027	−0.156	0.103	0.002	−0.017	0.243	– 0.117	– 0.042	1.000					

	(0.028)	(0.045)	(0.865)	(0.336)	(0.526)	(0.989)	(0.918)	(0.132)	(0.472)	(0.795)						
IL-6	0.282 (0.082)	0.490 **(0.001)**	0.368 (0.019)	0.148 (0.362)	0.284 (0.076)	0.204 (0.206)	0.198 (0.221)	0.623 **(<0.001)**	−0.012 (0.941)	0.168 (0.299)	0.058 (0.724)	1.000				
IL-8	0.024 (0.884)	0.324 (0.042)	0.169 (0.298)	0.173 (0.287)	0.110 (0.499)	0.159 (0.329)	0.391 (0.013)	−0.012 (0.941)	0.373 (0.018)	0.286 (0.074)	−0.082 (0.617)	0.330 (0.037)	1.000			
TNF-α€	0.135 (0.412)	0.043 (0.790)	0.341 (0.031)	0.373 (0.018)	0.457 **(0.003)**	0.155 (0.339)	0.177 (0.274)	0.228 (0.158)	0.044 (0.786)	0.251 (0.118)	−0.251 (0.118)	0.331 (0.037)	0.430 (0.006)	1.000		
VEGF	0.053 (0.749)	0.165 (0.308)	0.094 (0.563)	−0.11 (0.472)	0.119 (0.466)	0.188 (0.245)	0.040 (0.808)	0.257 (0.109)	0.147 (0.364)	0.032 (0.843)	0.233 (0.147)	0.309 (0.053)	0.170 (0.294)	0.046 (0.779)	1.000	
Surfprot	−0.212 (0.194)	0.045 (0.782)	0.080 (0.623)	0.176 (0.279)	0.246 (0.125)	0.030 (0.853)	0.246 (0.127)	−0.026 (0.876)	−0.066 (0.685)	0.232 (0.149)	0.062 (0.702)	−0.085 (0.600)	0.133 (0.413)	0.212 (0.189)	−0.197 (0.224)	1.000

Ang-2: Angiopoietin 2; CRP: C-reactive protein; ICH: intracerebral hemorrhage; IL-6: interleukin 6; IL-8: interleukin 8; MMP-1: matrix metalloproteinase 1; MMP-3: matrix metalloproteinase 3; MMP-9: matrix metalloproteinase 9; Pai-1 active: active Plasminogen Activator Inhibitor-1; Pai-1 total: total Plasminogen Activator Inhibitor-1; r: correlation coefficient; RAGE: Receptor for Advanced Glycation Endproducts; Surfprotd: Surfactant protein-D; TNF-α: tumor necrosis factor alpha; VEGF: vascular endothelial growth factor**.** Significance set to $p < 0.01$; bolded for identification.

Table A3. Multiple logistic regression for serum gonadal hormones and inflammatory proteins on dichotomized modified Rankin Scale (0–3 versus 4–6) at 6 months after intracerebral hemorrhage, adjusted for race, sex, age, body mass index, hypertension, hematoma location, hematoma volume, intraventricular hemorrhage.

	Point Estimate	95% Confidence Limits	p-Value
Estrogen, Day 1	0.995	(0.970, 1.020)	0.6785
Estrogen, Day 2	1.019	(0.986, 1.052)	0.2598
Estrogen, FC	1.078	(0.175, 6.629)	0.9357
Estrogen, LR	3.812	(0.727, 19.990)	0.1135
Progesterone, Day 1	2.980	(0.411, 21.614)	0.2800
Progesterone, Day 2	1.455	(0.153, 13.890)	0.7444
Progesterone, FC	1.157	(0.397, 3.376)	0.7893
Progesterone, LR	1.001	(0.570, 1.758)	0.9980
Testosterone, Day 1	4.723	(1.069, 20.858)	0.0405
Testosterone, Day 2	1.693	(0.589, 4.868)	0.3285
Testosterone, FC	0.129	(0.016, 1.032)	0.0536
Testosterone, LR	0.237	(0.053, 1.056)	0.0589
Ang-2, Day 1	1.002	(0.999, 1.004)	0.1594
Ang-2, Day 2	1.003	(0.999, 1.006)	0.1056
Ang-2, FC	8.561	(0.654, 112.112)	0.1018
Ang-2, LR	5.111	(0.568, 45.983)	0.1455
Pai-1 active, Day 1	1.000	(1.000, 1.000)	0.9964
Pai-1 active, Day 2	1.001	(1.000, 1.001)	0.0569
Pai-1 active, FC	4.290	(1.298, 14.176)	0.0169
Pai-1 active, LR	1.519	(0.556, 4.153)	0.4149
Pai-1 total, Day 1	1.000	(1.000, 1.000)	0.2689
Pai-1 total, Day 2	1.000	(1.000, 1.000)	0.8799
Pai-1 total, FC	71.055	(1.256, >999.999)	0.0384
Pai-1 total, LR	23.737	(1.541, 365.610)	0.0232
RAGE, Day 1	1.001	(1.000, 1.003)	0.1140
RAGE, Day 2	1.000	(1.000, 1.000)	0.3313
RAGE, FC	1.163	(0.499, 2.712)	0.7268
RAGE, LR	0.832	(0.295, 2.351)	0.7289
CRP, Day 1	1.000	(1.000, 1.000)	0.4851
CRP, Day 2	1.000	(1.000, 1.000)	0.0164
CRP, FC	3.175	(0.996, 10.119)	0.0507
CRP, LR	7.093	(1.210, 41.569)	0.0299
MMP-1, Day 1	1.000	(1.000, 1.000)	0.2395
MMP-1, Day 2	1.000	(1.000, 1.000)	0.1168
MMP-1, FC	10.178	(1.421, 72.890)	0.0209
MMP-1, LR	3.679	(0.722, 18.746)	0.1170
MMP-3, Day 1	1.000	(1.000, 1.000)	0.8373
MMP-3, Day 2	1.000	(1.000, 1.000)	0.4621
MMP-3, FC	0.592	(0.220, 1.592)	0.2989
MMP-3, LR	0.482	(0.112, 2.076)	0.3272
MMP-9, Day 1	1.000	(1.000, 1.000)	0.7853
MMP-9, Day 2	1.000	(1.000, 1.000)	0.9463
MMP-9, FC	2.161	(0.644, 7.257)	0.2123
MMP-9, LR	1.237	(0.374, 4.091)	0.7271
IL-6, Day 1	1.723	(1.046, 2.839)	0.0326

IL-6, Day 2	1.323	(1.049, 1.668)	0.0183
IL-6, FC	0.493	(0.187, 1.295)	0.1510
IL-6, LR	0.568	(0.275, 1.174)	0.1268
IL-8, Day 1	1.115	(0.954, 1.302)	0.1720
IL-8, Day 2	1.298	(0.934, 1.803)	0.1205
IL-8, FC	0.668	(0.191, 2.343)	0.5290
IL-8, LR	0.844	(0.295, 2.417)	0.7523
TNF-α, Day 1	1.601	(0.934, 2.744)	0.0872
TNF-α, Day 2	1.759	(0.828, 3.738)	0.1422
TNF-α, FC	0.649	(0.054, 7.845)	0.7338
TNF-α, LR	0.366	(0.036, 3.716)	0.3951
VEGF, Day 1	1.006	(0.974, 1.039)	0.7122
VEGF, Day 2	1.001	(0.966, 1.037)	0.9725
VEGF, FC	0.587	(0.033, 10.546)	0.7180
VEGF, LR	0.562	(0.082, 3.877)	0.5589
Surfprot, Day 1	0.962	(0.811, 1.141)	0.6564
Surfprot, Day 2	0.923	(0.764, 1.114)	0.4030
Surfprot, FC	0.016	(<0.001, 6.648)	0.1781
Surfprot, LR	0.078	(0.002, 3.629)	0.1927

Ang-2: Angiopoietin 2; CRP: C-reactive protein; Day 1: serum concentration one day after ICH; Day 2: serum concentration two days after ICH; FC: Fold Change = Day 2/Day1; IL-6: interleukin 6; IL-8: interleukin 8; LR: Log ratio = Log2(Day 2/Day 1); MMP-1: matrix metalloproteinase 1; MMP-3: matrix metalloproteinase 3; MMP-9: matrix metalloproteinase 9; Pai-1 active: active Plasminogen Activator Inhibitor-1; Pai-1 total: total Plasminogen Activator Inhibitor-1; RAGE: Receptor for Advanced Glycation Endproducts; Surfprotd: Surfactant protein-D; TNF-α: tumor necrosis factor alpha; VEGF: vascular endothelial growth factor.

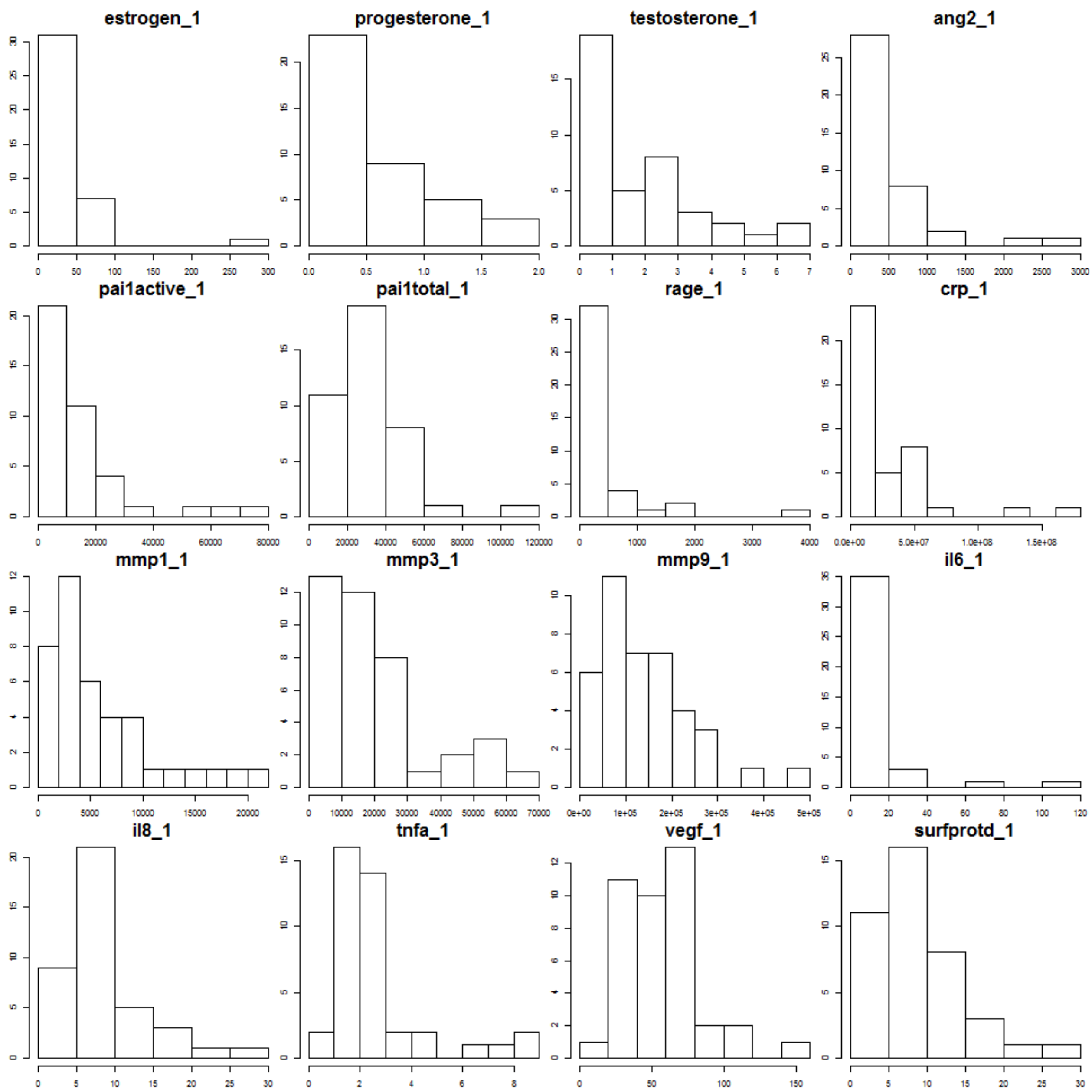

Figure S1. Histograms of Day 1 gonadal hormone and inflammatory protein serum concentrations after ICH. X-axis: Day 1; Y-axis: percentage of samples. Ang-2: Angiopoietin 2 (pg/mL); Day 1: serum concentration one day after ICH; CRP: C-reactive protein (mg/dL); Estrogen (pg/mL); ICH: intracerebral hemorrhage; IL-6: interleukin 6 (pg/mL); IL-8: interleukin 8 (pg/mL); MMP-1: matrix metalloproteinase 1 (ng/mL); MMP-3: matrix metalloproteinase 3 (ng/mL); MMP-9: matrix metalloproteinase 9 (ng/mL); Pai-1 active: active Plasminogen Activator Inhibitor-1 (IU/mL); Pai-1 total: total Plasminogen Activator Inhibitor-1 (IU/mL); Progesterone (ng/mL); RAGE: Receptor for Advanced Glycation Endproducts (pg/mL); Surfprotd: Surfactant protein-D (units not specified); Testosterone (ng/dL); TNF-α: tumor necrosis factor alpha (pg/mL); VEGF: vascular endothelial growth factor (pg/mL).

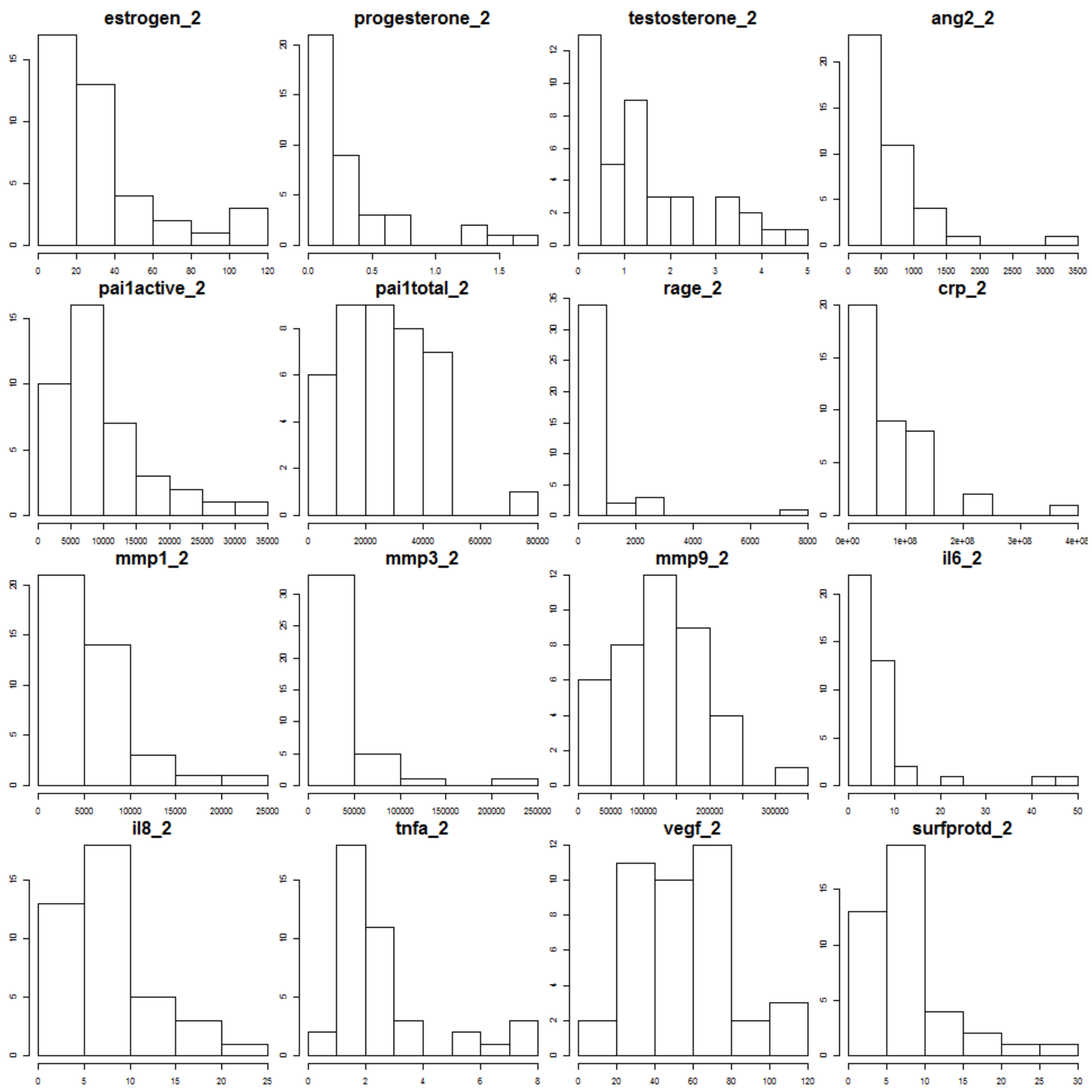

Figure S2. Histograms of Day 2 gonadal hormone and inflammatory protein serum concentrations after ICH. X-axis: Day 2; Y-axis: percentage of samples. Ang-2: Angiopoietin 2 (pg/mL); CRP: C-reactive protein (mg/dL); Day 2: serum concentration two days after ICH; Estrogen (pg/mL); ICH: intracerebral hemorrhage; IL-6: interleukin 6 (pg/mL); IL-8: interleukin 8 (pg/mL); MMP-1: matrix metalloproteinase 1 (ng/mL); MMP-3: matrix metalloproteinase 3 (ng/mL); MMP-9: matrix metalloproteinase 9 (ng/mL); Pai-1 active: active Plasminogen Activator Inhibitor-1 (IU/mL); Pai-1 total: total Plasminogen Activator Inhibitor-1 (IU/mL); Progesterone (ng/mL); RAGE: Receptor for Advanced Glycation Endproducts (pg/mL); Surfprotd: Surfactant protein-D (units not specified); Testosterone (ng/dL); TNF-α: tumor necrosis factor alpha (pg/mL); VEGF: vascular endothelial growth factor (pg/mL).

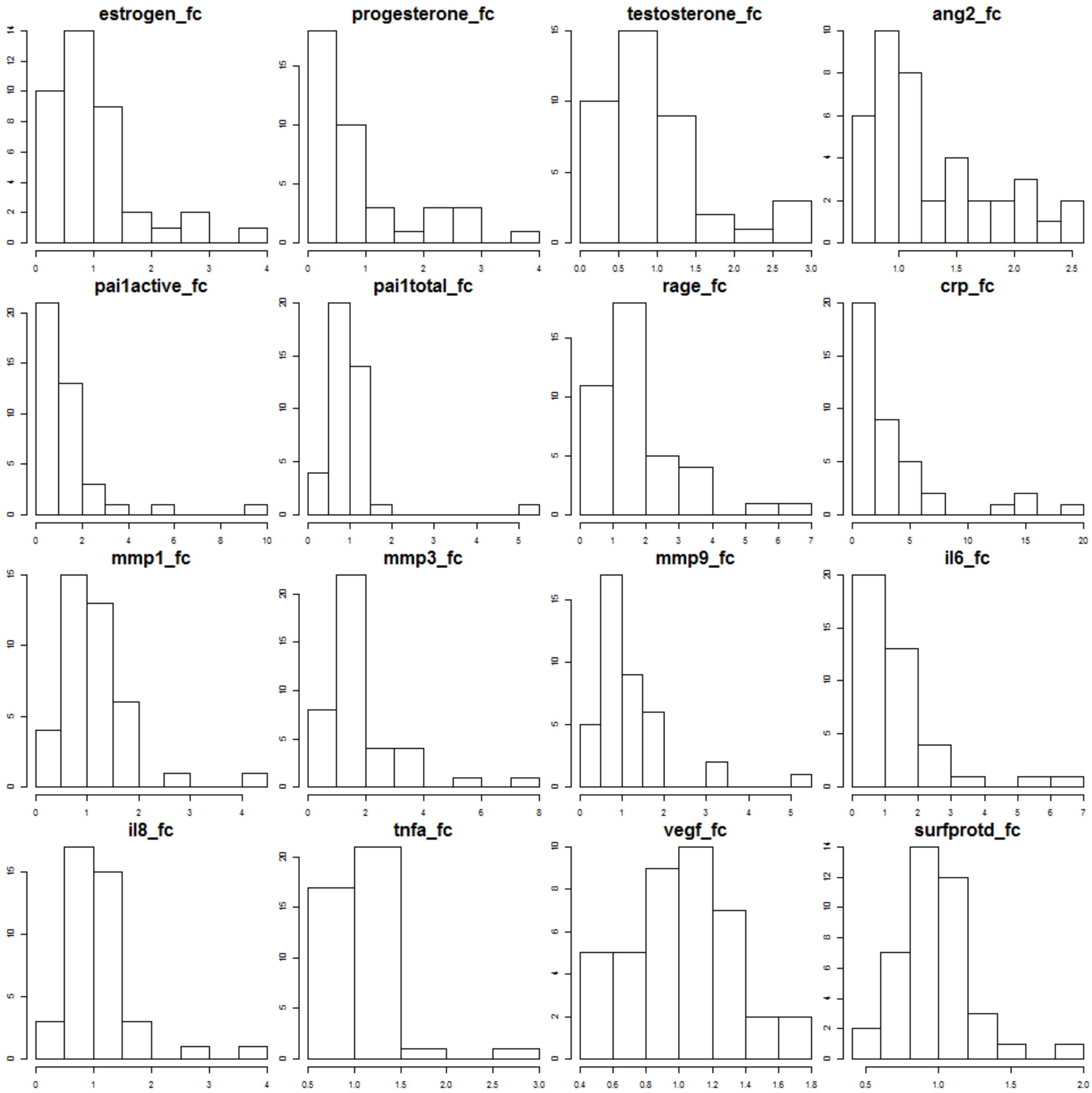

Figure S3. Histograms of FC for gonadal hormone and inflammatory protein serum concentrations after ICH. X-axis: FC: Fold Change; Y-axis: percentage of samples. Ang-2: Angiopoietin 2; CRP: C-reactive protein; Day 1: serum concentration one day after ICH; Day 2: serum concentration two days after ICH; FC: Fold Change = Day 2/Day1; Estrogen; ICH: intracerebral hemorrhage; IL-6: interleukin 6; IL-8: interleukin 8; MMP-1: matrix metalloproteinase 1; MMP-3: matrix metalloproteinase 3; MMP-9: matrix metalloproteinase 9; Pai-1 active: active Plasminogen Activator Inhibitor-1; Pai-1 total: total Plasminogen Activator Inhibitor-1; Progesterone; RAGE: Receptor for Advanced Glycation Endproducts; Surfprotd: Surfactant protein-D; Testosterone; TNF-α: tumor necrosis factor alpha; VEGF: vascular endothelial growth factor.

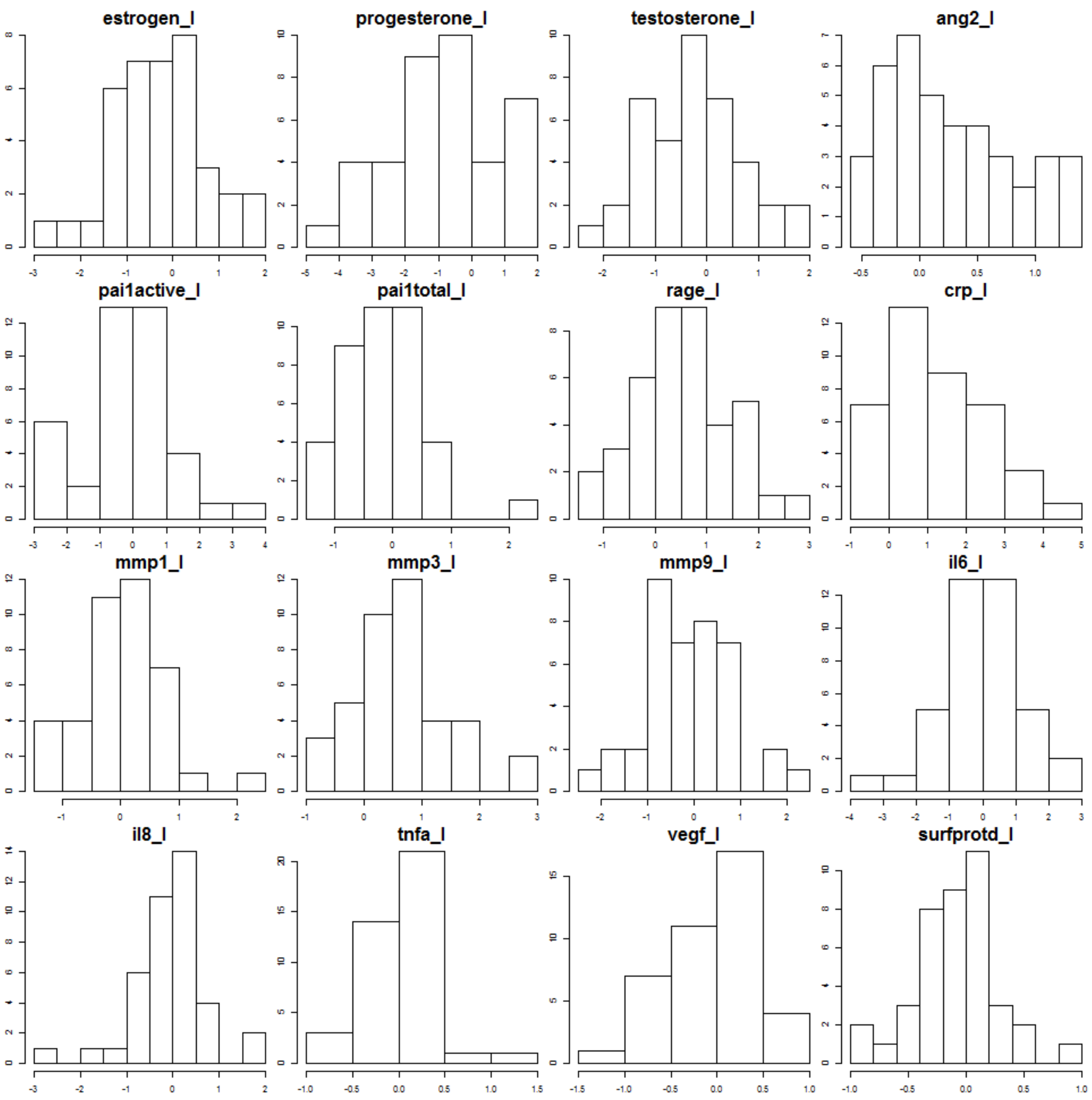

Figure S4. Histograms of LR for gonadal hormone and inflammatory protein serum concentrations after ICH. X-axis: LR; Y-axis: percentage of samples. Ang-2: Angiopoietin 2; CRP: C-reactive protein; Day 1: serum concentration one day after ICH; Day 2: serum concentration two days after ICH; Estrogen; ICH: intracerebral hemorrhage; IL-6: interleukin 6; IL-8: interleukin 8; LR: log ratio= Log2(Day 2)/Day 1); MMP-1: matrix metalloproteinase 1; MMP-3: matrix metalloproteinase 3; MMP-9: matrix metalloproteinase 9; Pai-1 active: active Plasminogen Activator Inhibitor-1; Pai-1 total: total Plasminogen Activator Inhibitor-1; Progesterone; RAGE: Receptor for Advanced Glycation Endproducts; Surfprotd: Surfactant protein-D; Testosterone; TNF-α: tumor necrosis factor alpha; VEGF: vascular endothelial growth factor
